# *Helicobacter pylori*, gastric cancer and socioeconomic factors in an urban area: Evaluating the strengths and limitations of spatial analysis

**DOI:** 10.1016/j.pmedr.2025.103138

**Published:** 2025-06-13

**Authors:** Stephanie Strobl, Giovenale Moirano

**Affiliations:** a Institute of Pathology of the University Medical Center Mainz, Johannes Gutenberg-University, Mainz, Germany; b Department of Medical Sciences, University of Turin and CPO-Piemonte, Torino, Italy

**Keywords:** Spatial epidemiology, *Helicobacter pylori*, Gastric cancer, Geospatial analysis, Disease distribution, Prevention

## Abstract

**Objective:**

Spatial epidemiology provides a valuable opportunity to utilize routinely collected clinical data for analyzing disease distribution. This study evaluates the potential of such data to model the geographical distribution of *Helicobacter pylori* (*H. pylori*) colonization and gastric cancer in Mainz, Germany. We aimed to assess the feasibility of spatial statistical analyses of clinical routine data, identify factors influencing *H. pylori* colonization, and investigate whether *H. pylori* colonization and gastric cancer share common spatial patterns and risk factors relevant for prevention strategies.

**Methods:**

Data on *H. pylori* colonization was extracted from routine gastric biopsy reports (2008–2019), while gastric cancer cases were derived from local cancer registry data (2019–2022). Geospatial data and socioeconomic variables were integrated into generalized linear mixed-effects models (GLMMs) to explore their associations with the diseases. The Moran's I statistic was used to assess spatial autocorrelation.

**Results:**

Among 19,727 biopsies analyzed, 24.7 % were *H. pylori*-positive, with colonization varying widely across districts (10.7 %–38.9 %). Significant associations were found with unemployment rates, household size, and foreign or immigrant background populations. In contrast, the GLMM for gastric cancer revealed no significant predictors, likely due to low case numbers (108 cases) and limited variation across districts. Nonetheless, the observed association between *H. pylori* and gastric cancer aligns with established literature.

**Conclusions:**

This study demonstrates the potential of routine data in spatial epidemiology for identifying at-risk populations. While challenges remain, particularly for rarer diseases, this approach provides valuable insights into disease distributions and can support targeted prevention strategies.

## Highlights

• Spatial study on *Helicobacter pylori* and gastric cancer using socioeconomic data.
• *Helicobacter pylori* shows spatial variation linked to socioeconomic conditions.
• Gastric cancer shows no spatial clustering, reflecting rarity and complexity.
• Results support spatial tools for cancer risk assessment and prevention planning.

## 1 Introduction

The connection between spatial epidemiology and pathology may not be immediately apparent. However, routine pathological diagnostics generate vast amounts of data daily, with hundreds of diagnoses recorded and stored electronically, including patient addresses. Despite this, spatial analyses rarely incorporate pathology-derived data, leaving a rich and underutilized resource largely untapped—even though diagnoses can be automatically extracted through text-mining and linked to geocoded patient addresses for spatial analysis of disease patterns.

By applying text-mining methodologies to pathological reports, specific diagnoses can be extracted and linked to geocoded patient addresses, enabling spatial analysis of disease patterns and potential risk factors. *Helicobacter pylori* (*H. pylori*) colonization and gastric cancer are compelling examples, given their established link and relevance for targeted prevention.

*H. pylori* itself is one of the most prevalent organisms in the human microbiome, colonizing up to 50 % of the global population (Alzahrani et al., 2014). Risk factors for *H. pylori* infection include densely populated environments (Mendall et al., 1992), poor sanitation (Diaconu et al., 2017), intra-household transmission (Stone et al., 2000; Bastos et al., 2013), maternal infection (Weyermann et al., 2009), specific diets (Razuka-Ebela et al., 2020), and genetic susceptibility (Malaty et al., 2000; Mayerle et al., 2013). Among these, low socioeconomic status (SES)—especially during childhood—is considered the strongest determinant of infection risk (Malaty and Graham, 1994).

*H. pylori* colonizes the gastric epithelium, where it can induce chronic inflammation of the gastric mucosa. This inflammation plays a pivotal role in the pathogenesis of several conditions, including duodenal and gastric ulcers (affecting 1–10 % of infected individuals), mucosa-associated lymphoid-tissue lymphomas (<0.01 % of infected individuals), and gastric cancers (0.1–3 % of infected individuals) (McColl, 2010). Additional risk factors for gastric cancer include advanced age, male sex, smoking, obesity, dietary habits, certain medications, genetic predispositions, and infections (Thrift et al., 2023).

This study investigates whether routinely collected diagnostic data on *H. pylori* colonization and gastric cancer can be effectively used for spatial epidemiological analysis. Specifically, we assess their geographical distribution, identify associated risk factors, and evaluate the potential of spatial analysis as a tool to support risk stratification and targeted prevention strategies.

Importantly, this study takes a novel approach by leveraging routinely collected, real-world diagnostic data—rather than curated research datasets—thus demonstrating the feasibility of integrating spatial analysis into everyday clinical workflows. While *H. pylori* serves as a primary focus, this method can be extended to other conditions with known or suspected spatial variation, such as certain infectious and occupational diseases.

## 2 Subjects and methods

### 2.1 Data source

Patient data was obtained from routine pathological diagnostics at the Institute of Pathology, University Medical Center Mainz (hereafter referred to as the Institute of Pathology). The Institute of Pathology is the sole pathological institute in the region, processing approximately 50,000 patient samples annually from the University Medical Center Mainz, as well as from smaller hospitals and doctors' offices in the surrounding area. Since there are no competing pathology institutes locally, and most diagnostic biopsies from city residents are processed here, the study population is considered broadly representative of individuals who regularly seek medical care in Mainz. However, as asymptomatic individuals are unlikely to undergo biopsy, selection bias may exist, potentially underestimating colonization in less symptomatic or undiagnosed cases.

Areal data, including city and district boundaries and all independent variables, was obtained from the Statistics Office of the Citizen Services Department of the City of Mainz (*Statistikstelle des Bürgeramts der Stadt Mainz*). Mainz is a medium-sized city in central Germany with approximately 225,000 inhabitants, located 50 km from Frankfurt am Main in the Rhine-Main region. The city is divided into 15 suburbs and 65 districts.

### 2.2 Case definitions

Data on *H. pylori* colonization was obtained through text-mining of all gastric biopsy reports stored in the pathological reporting software, PathoPro (IFMS, Germany), using an in-house developed, C-based script. The search focused on *H. pylori* status, which pathologists are required to report. To reflect the most recent status and avoid duplication, only the latest biopsy per patient was considered. The analysis timeframe (2008–2019) was selected to ensure stable biopsy rates and standardized in-house documentation.

With regard to gastric cancer cases, we used the data reported to the Cancer Registry of Rhineland-Palatinate (*Krebsregister Rheinland-Pfalz*) referred to the 2019–2022 period. In Germany, reporting all cancer diagnoses to a registry is legally required (Rheinland-Pfalz, 2007). The Institute of Pathology submits all confirmed diagnoses, including ICD-O-3 site codes, which are used to identify tumors of gastric origin (Supplementary Table 1). The timeframe for studying gastric cancer cases was restricted to the years 2019–2022 for two key reasons. First, the reporting process at the Institute of Pathology was standardized and streamlined in collaboration with the local cancer registry in mid-2018, making 2019 the first year with fully standardized reporting. Second, at the time of data analysis, the ICD-O-3 coding procedure at the Institute of Pathology had not yet been completed for the year 2023. Lastly, as long-term exposure to *H. pylori* is necessary to potentially develop a gastric neoplasm, aforementioned differences in the timeframes of data collection, which are caused by the methods of data collection, were accepted.

### 2.3 Geocoding

Both patients who underwent stomach biopsies and gastric cancer cases were geocoded based on their permanent addresses using the geopy package and the Nominatim geocoder in Python. After geocoding, patients were assigned to their respective districts based on the GPS coordinates falling within the multipolygon data of the city of Mainz.

While Nominatim provides sufficient address-level accuracy for urban areas like Mainz, limitations may exist for incomplete, outdated, or rural addresses. Geocoding failures were rare and occurred only when address fields were missing; such cases were excluded from spatial analyses (*n* = 24).

### 2.4 Independent variables

Independent variables examined in relation to both H.pylori prevalence and gastric cancer incidence included proxies for socioeconomic status (SES), the residential environment, and differences in genetic susceptibility (Table 1). With regard to the latter, the foreign population is defined as non-German nationals, while the population with an immigrant background is defined—according to the local statistics bureau—as persons who either hold one or more nationalities in addition to German nationality, were born abroad, or have at least one parent who was born abroad.

**Table 1:** Independent variables included in the statistical modeling of *Helicobacter pylori* colonization and gastric cancer incidence among residents of Mainz, Germany (2008–2022).

Independent Variables	Unit of Analysis	Dependent Variables	
		*H. pylori*-positivity of gastric biopsies	SIR for gastric cancer
Proportion of unemployed population	per 5 % increase	X	X
Mean rent per square meter	per € increase	X	X
Proportion of industrial/commerce area	per 5 % increase	X	X
Population density [inhabitants/ km^2^]	per 10 persons	X	–
Proportion of households with >5 residents	per 10 % increase	X	–
Proportion of foreign or immigrant background population	per 10 % increase	X	X
Unemployment among foreign or immigrant background population	per 5 % increase	X	X
Proportion of *H. pylori*-positive gastric biopsies	per 10 % increase	–	X
Standardized incidence ratio of lung cancer	–	–	X

X = variable included in the respective model.

SIR = standardized incidence ratio.

In addition, for modeling *H. pylori***-positivity** in gastric biopsies, factors influencing the likelihood of acquiring and transmitting infectious diseases were included in the analysis such as population density and the proportion of households with greater than five residents (Table 1).

For modeling the Standardized Incidence Ratio (SIR) of gastric cancer, known risk factors for this disease were included in the analysis. In addition to *H. pylori* colonization, we included the SIR for lung cancer as a proxy indicator for tobacco consumption, given that smoking is an established risk factor for gastric cancer (National Cancer Institute, 2023). This approach allows for partial adjustment for district-level smoking patterns in the absence of individual smoking data.

The SIR for gastric and lung cancer patients were calculated from the number of cancer cases diagnosed at the Institute of Pathology from 2019 to 2022, classified according to ICD-O-3 Site Codes (Supplementary Tables 1 and 2), and the age- and sex-stratified data provided by the local statistics bureau.

The study was approved by a positive vote from the Ethics Committee of the State Medical Association of Rhineland-Palatinate (*Ethikkommission der Landesärztekammer Rheinland-Pfalz*, protocol number: 2021–15741-retrospektiv, date of adoption: 13 April 2021).

### 2.5 Statistical analyses

To assess spatial patterns, we applied Moran's I statistic to evaluate spatial autocorrelation in the distribution of *H. pylori* and gastric cancer incidence across districts. Moran's I tests for spatial autocorrelation by comparing values across neighboring geographic units against the null hypothesis of random spatial distribution (Moran, 1950). Positive values indicate clustering, negative values indicate dispersion (Rogerson and Jacquez, 2016). Since no significant spatial autocorrelation was detected in either outcome, conditional autoregressive models were not applied.

To analyze *H. pylori* colonization, a binomial regression model was fitted using a Generalized Linear Mixed-Effects Model (GLMM), with district included as a random effect. All estimates were adjusted for the proportion of residents over age 65 and the proportion of female residents.

GLMMs were selected due to their flexibility in handling grouped data structures (i.e., districts) and their ability to account for unobserved heterogeneity via random effects. While spatial regression models can be useful, they are most appropriate when spatial autocorrelation is present—which was not the case in this dataset.

To model gastric cancer incidence, a Poisson GLMM was used, appropriate for rare outcomes. District was again treated as a random effect, and the expected number of cases (based on district age and sex composition) was included as an offset for modeling the incident cases of gastric cancer.

All models were implemented in R using the glmer function from the lme4 package and the moran.mc function from the spdep package.

## 3 Results

### 3.1 Descriptive analyses

In the city of Mainz, 19,727 gastric biopsies from permanent residents were analyzed at the Institute of Pathology between 2008 and 2019. Throughout the study period, the number of gastric biopsies processed at the Institute remained relatively stable, with slight increases observed in the years 2011–2013 and 2018/2019.

Out of 19,727 gastric biopsies, 4878 cases of *H. pylori* colonization were detected, representing 24.7 % of all gastric biopsies.

Regarding gastric cancer, there were 108 new diagnoses between 2019 and 2022. Over the years, the number of diagnosed gastric cancer cases remained relatively stable, with a slight decline observed in 2022.

### 3.2 Spatial analysis of *H. pylori*

**Fig. 1:**
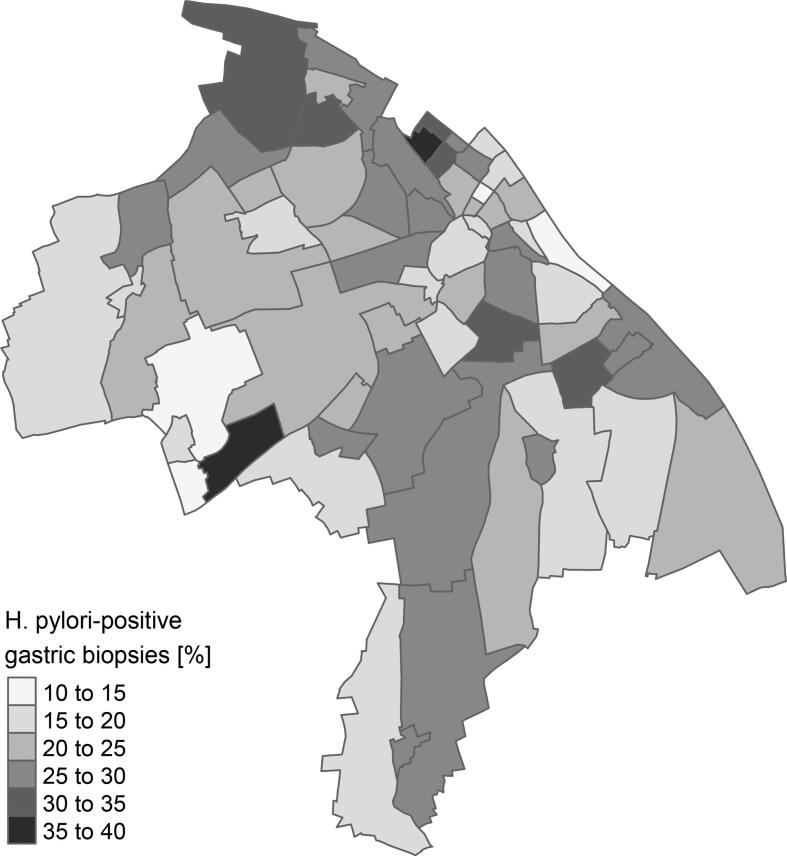
Spatial distribution of the proportion of gastric biopsies testing positive for *Helicobacter pylori* among residents of Mainz, Germany (2008–2019).

The distribution of *H. pylori***-positive** gastric biopsies across districts in Mainz was heterogeneous, with values ranging from 10.7 % to 38.9 % (Fig. 1).

The Moran's I statistic, accounting for a GLMM model of the proportion of *H. pylori*-positive biopsies, showed no evidence of spatial autocorrelation (*p*-value 0.21).

In regression analysis, variables that significantly explained the distribution of *H. pylori*-positive gastric biopsies included the unemployment rate, the proportion of households with more than five residents, the proportion of foreign residents or those with an immigrant background, and the unemployment rate among foreign residents and those with an immigrant background (Table 2). A 5 % increase in the unemployment rate corresponded to a 2.63-fold increase in the odds of *H. pylori*-positivity in gastric biopsies, while a 10 % increase in households with more than five residents led to a 1.22-fold increase (Table 2). Additionally, a 10 % increase in unemployment among the foreign and immigrant background population resulted in a 2.65-fold increase in *H. pylori* positivity (Table 2). The most striking finding was a relative increase of 10 % in the proportion of the foreign and immigrant background population, which increased the odds of *H. pylori*-positive biopsies by 13.56 (Table 2).

**Table 2:** Adjusted odds ratios from generalized linear mixed-effects model assessing factors associated with *H. pylori* positivity in gastric biopsies among residents of Mainz, Germany (2008–2019).

Variable	Adjusted Odds Ratio (OR)	95 % Confidence Interval (CI)
Unemployed population (%)	2.63	[1.97, 3.55]
Rent per m^2^ (€)	0.98	[0.9, 1.07]
Industrial/commercial land (%)	1.06	[0.66, 1.72]
Population density (per 10 inhabitants/ km^2^]	0.99	[0.43, 2.27]
Households with >5 residents (%)	1.22	[1.14, 1.29]
Foreign or immigrant background (%)	13.56	[8.11, 22.99]
Unemployment among foreign or immigrant background (%)	2.65	[1.31, 5.41]

OR = odds ratio, adjusted for district-level proportion of women and residents over age 50.

CI = confidence interval.

**Fig. 2:**
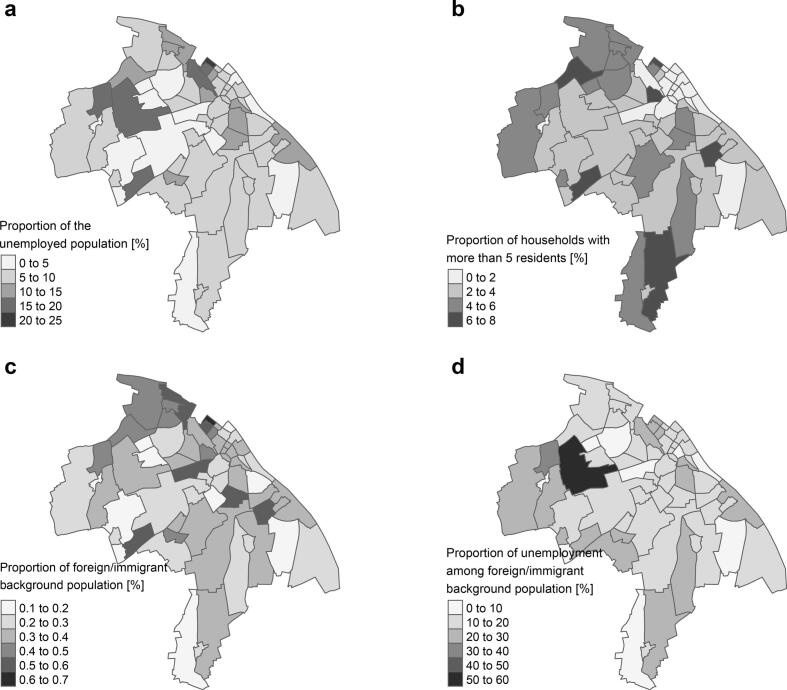
Key district-level socioeconomic and demographic variables associated with *Helicobacter pylori* positivity in gastric biopsies among residents of Mainz, Germany (2008–2019). (a) Unemployed population (%), (b) Households with more than five residents (%), (c) Foreign or immigrant background population (%), (d) Unemployment among foreign or immigrant population (%).

A graphical representation of the independent variables influencing the distribution of *H. pylori***-positive** gastric biopsies according in the adjusted model is shown in Fig. 2.

### 3.3 Spatial analysis of gastric cancer

**Fig. 3:**
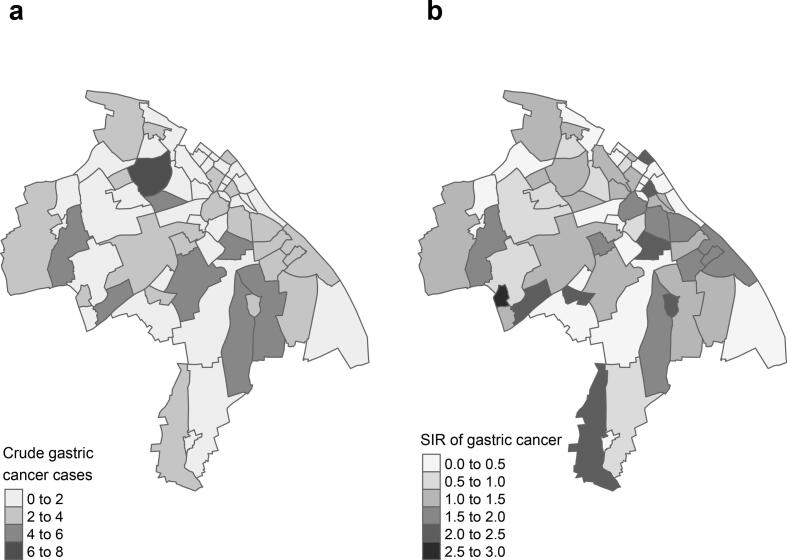
Spatial distribution of newly diagnosed gastric cancer cases among residents of Mainz, Germany (2019–2022). (a) Crude case numbers by district, (b) Standardized incidence ratios (age- and sex-adjusted) by district.

The distribution of gastric cancer cases across districts of Mainz was also heterogeneous, with crude case numbers ranging from 0 to 8 (Fig. 3).

The Moran's I statistic of the standardized incidence ratios for gastric cancer showed no evidence of spatial autocorrelation, indicating a random spatial distribution (*p*-value: 0.88).

When modeling the standardized incidence ratios for gastric cancer, none of the variables included in the GLMM sufficiently explained the distribution of gastric cancer cases (Table 3).

**Table 3:** Relative risks from generalized linear mixed-effects model for district-level gastric cancer across city districts in Mainz, Germany (2019–2022).

Variable	Relative Risk (RR)	95 % Confidence Interval (CI)
Unemployed population (%)	0.87	[0.3, 2.57]
Rent per m^2^ (€)	1.07	[0.8, 1.43]
Industrial/commercial land (%)	1.04	[0.22, 3.92]
Foreign or immigrant background (%)	1.44	[0.15, 12.46]
Unemployment among foreign/immigrant background (%)	0.93	[0.13, 5.11]
Proportion of *H. pylori*-positive gastric biopsies	2.07	[0.26, 15.53]
Lung cancer incidence (SIR)	0.87	[0.66, 1.1]

RR = relative risk.

CI = confidence interval.

SIR = standardized incidence ratio. Models adjusted for district-level age and sex distributions.

## 4 Discussion

In the population of Mainz, the proportion of *H. pylori***-positive** gastric biopsies varied greatly, ranging from 10.7 % to 38.9 % across city districts. These numbers are comparable to colonization rates reported in Germany. The German National Health Survey 1998 (*Bundes-Gesundheitssurvey 1998*, BGS98) found that 40 % of adults tested positive for antibodies against *H. pylori* (Seher et al., 2000; Gastritis, 2013), while the Epidemiological Study on the Chances of Prevention, Early Detection, and Optimized Therapy of Chronic Diseases in the Elderly (*Epidemiologische Studie zu Chancen der Verhütung, Früherkennung und optimierten Therapie chronischer Erkrankungen in der älteren Bevölkerung*, ESTHER) reported a seropositivity rate of 52.6 % among adults (Gao et al., 2009). Although the proportion of *H. pylori***-positive** gastric biopsies in Mainz was slightly lower (mean 23.78 %, median 24.01 %), these differences can be attributed to the method of data collection: the aforementioned studies assessed seropositivity (i.e., lifetime exposure), while our study captured real-time colonization. Thus, data from routine pathological diagnostics appear to accurately reflect active colonization patterns in Germany. The observed increases in biopsy numbers during certain years may be due to changes in submission patterns from local hospitals and outpatient facilities, as supported by internal documentation.

The ecological regression analysis identified the proportion of foreign nationals and individuals with an immigrant background as the most prominent variable explaining *H. pylori* distribution (Table 2). These findings align with international data showing *H. pylori* prevalence differences by geography and ethnicity—ranging from 75 to 83 % in Latin America, 39.6 % in Japan, to 17.1 % in the USA (Kao et al., 2016), and elevated prevalence among Americans with African or Hispanic ancestry (Everhart et al., 2000; Epplein et al., 2011). Our study adds to this body of evidence, showing similar patterns on a local scale.

Notably, the strong association observed—specifically, an odds ratio of 13.56 for districts with a higher proportion of individuals with an immigrant background—highlights a potential focus for public health action. This may reflect disparities in socioeconomic conditions, healthcare access, or early-life exposures. While our data are aggregated, future studies should examine whether specific subgroups within the immigrant background population are disproportionately affected, to support more targeted screening strategies.

The regression analysis also confirmed known social determinants of *H. pylori* infection. Higher unemployment rates—both in the general population and specifically among foreign nationals—were associated with *H. pylori* colonization (Table 2). This is consistent with previous studies showing that *H. pylori* prevalence correlates with lower socioeconomic status (SES), as seen in the BGS98 and the German Health Interview and Examination Survey for Children and Adolescents (*Kinder- und Jugendgesundheitssurvey*, KIGGS) studies (Seher et al., 2000; Gastritis, 2013). Risk factors such as overcrowded housing and close contact with infected individuals (Mendall et al., 1992; Stone et al., 2000; Bastos et al., 2013) were also supported by our findings, as households with more than five residents had increased odds of *H. pylori* positivity (Table 2).

This ecological approach to modeling *H. pylori* colonization supports and extends existing literature, demonstrating the utility of routinely collected diagnostic data for spatial health analysis. Moreover, the findings may inform actionable public health strategies, such as implementing broad, non-invasive screening programs in high-prevalence districts—potentially via general practitioners or hospitals.

In contrast, the spatial analysis for gastric cancer yielded less conclusive results. Although gastric cancer is known to show geographic variability (Polk and Peek, 2010) and is linked to *H. pylori* colonization (Etemadi et al., 2020), our models did not detect a significant association between these variables (Table 3). Similarly, the standardized incidence ratio (SIR) for lung cancer—a proxy for smoking patterns—was not associated with gastric cancer distribution (Table 3).

Several factors may explain the lack of significant spatial associations for gastric cancer in our analysis. Most notably, the small number of gastric cancer cases (*n* = 108) and low counts per district limited statistical power and reduced variation across geographic units, likely contributing to a Type II error. Additionally, the confined geographical area of a single city may not capture sufficient spatial variability compared to larger-scale regional studies. The timeframe of available data may also be too short to detect long-latency effects, such as the progression from *H. pylori* infection to gastric cancer. Moreover, the multifactorial and long-term nature of carcinogenesis adds complexity, as it involves interactions between genetic, lifestyle, and environmental factors that are not easily captured in routine datasets. While the relative risk estimate (RR: 2.07) for *H. pylori*-positive biopsies pointed in the expected direction, the wide confidence intervals (95 % CI, 0.26–15.53) indicate low precision. To address these challenges, future studies should consider multi-city collaborations or pooling regional data to increase case numbers and enhance analytical robustness.

In addition, individual-level risk factors that influence gastric carcinogenesis—such as family history, hereditary cancer syndromes (e.g., familial adenomatous polyposis), specific dietary habits, Epstein-Barr virus infection, or environmental and occupational exposures (e.g., radiation) (National Cancer Institute, 2023)—were not captured in our dataset. This underscores a broader challenge in ecological studies: the risk of ecological fallacy. That is, associations observed at the population level may not apply to individuals. Variables such as immigrant background may serve as proxies for unmeasured individual-level factors, including genetics, diet, or lifestyle, which could not be directly accounted for in our analysis.

It is also worth noting that not all *H. pylori*-infected individuals are identified through pathological diagnostics. Many are asymptomatic and may not undergo biopsy, while other diagnostic approaches—such as urea breath tests or serological assays—are not typically reported to pathology institutes (Fischbach et al., 2016). Therefore, the dataset likely underrepresents the full burden of infection.

Taking these limitations into accounts, this study has demonstrated that the distribution of *H. pylori*-positive biopsies can be effectively analyzed using data from routine pathological diagnostics. The heterogeneity in *H. pylori* colonization across the study area was successfully explained by the proportion of the foreign and immigrant background population, underscoring the value of spatial analysis in revealing meaningful patterns in population health. Conversely, the spatial model did not explain the distribution of gastric cancer cases, likely due to small case numbers or long latency that limited model performance.

Despite these limitations, spatial statistical models offer valuable insights to analyze patological records data by adding a geographical dimension to routinely collected medical records. Their major advantage lies in highlighting underlying factors that shape observed distributions. While small case numbers can restrict these models' ability to pinpoint geographic risk factors for rarer diseases, they remain a strong complement to conventional studies using larger datasets.

## 5 Conclusion

The successful analysis of *H. pylori* positivity in routine gastric biopsies highlights the potential of spatial statistical models and should encourage further exploration and refinement of these methods. By modeling disease distributions and determinants in a geographical context, as demonstrated here, researchers can advance the development of these models and gain deeper insights into both their strengths and limitations, ultimately improving their application in epidemiological research and our understanding of disease distribution.

Importantly, the methodology is scalable and currently being operationalized through the development of an automated tool that screens pathology reports daily for tumor diagnoses. These data are fed into an application for real-time descriptive and statistical analysis. In the future, the system will be expanded to include all diagnoses made at the Institute of Pathology Mainz, enabling broader disease surveillance and continuous spatial monitoring. As part of this ongoing expansion, the integration of external environmental datasets—such as locally collected air pollution or water quality data—is also envisaged. Incorporating these dimensions will further refine risk factor analyses and enhance the public health relevance of spatial epidemiological models.

## CRediT authorship contribution statement

**Stephanie Strobl:** Writing – original draft, Visualization, Project administration, Methodology, Investigation, Formal analysis, Conceptualization. **Giovenale Moirano:** Supervision, Methodology, Investigation, Conceptualization.

## Ethics statement

All analyses were conducted in accordance with applicable laws and institutional guidelines. The study received ethical approval from the Ethics Committee of the State Medical Association of Rhineland-Palatinate (*Ethikkommission der Landesärztekammer Rheinland-Pfalz*; protocol number: 2021–15,741-retrospektiv, approval date: 13 April 2021). Furthermore, all measures were taken to ensure the protection of participants' privacy and confidentiality.

## Funding

Stephanie Strobl was supported by the TransMed Fellowship of the UCT Mainz (Universitäres Centrum für Tumorerkrankungen Mainz) for Clinician Scientists as part of the overarching project, *Using Pathological Routine Diagnostics to Identify Populations at Risk for Gastrointestinal Cancers – A Spatial Ecological Study*. All other authors did not receive funding related to this work.

## Declaration of competing interest

The authors declare that they have no known competing financial interests or personal relationships that could have appeared to influence the work reported in this paper.

## Data Availability

All aggregated patient data analyzed in this study is included within this article. Spatial data was obtained from the Statistics Office of the Citizen Services Department of the City of Mainz (*Statistikstelle des Bürgeramts der Stadt Mainz*) and is available upon request from this office. Supplementary material is available and includes a detailed listing of the ICD-O-3 anatomical localization codes used for case identification. No additional data is available upon request due to confidentiality restrictions, as it contains individual patient data.

## Appendix A Supplementary data

Supplementary Tables Image 1
