# Supplementary material for: Helicobacter pylori, gastric cancer and socioeconomic factors in an urban area: Evaluating the strengths and limitations of spatial analysis
Source: Prev Med Rep. 2025 Jun 13;56:103138. doi: 10.1016/j.pmedr.2025.103138 (PMC12212250; doi:10.1016/j.pmedr.2025.103138)
Supplement: Supplementary file 1 — Supplementary Tables [file mmc1.docx]

**Supplementary Information**

**Supplementary Table 1. International Classification of Diseases for Oncology, Third Edition (ICD-O-3) site codes for anatomical subregions of the stomach used in the classification of gastric tumors**

| ICD-O-3 Code | Anatomical Subregions of the Stomach |
| --- | --- |
| C16.0 | Cardia, not otherwise specified |
| C16.1 | Fundus of the stomach |
| C16.2 | Body oft he stomach |
| C16.3 | Gastric antrum |
| C16.4 | Pylorus |
| C16.5 | Lesser curvature of stomach, not otherwise specified |
| C16.6 | Greater curvature of stomach, not otherwise specified |
| C16.8 | Overlapping lesion of stomach |
| C16.9 | Stomach, not otherwise specified |

**Footnote:** ICD-O-3 = International Classification of Diseases for Oncology, Third Edition. Source: (1)

**Supplementary Table 2. International Classification of Diseases for Oncology, Third Edition (ICD-O-3) site codes for anatomical subregions of the lung used in the classification of pulmonary tumors**

| ICD-O-3 Code | Anatomical Subregions of the Lung |
| --- | --- |
| C34.0 | Main bronchus |
| C34.1 | Upper lobe |
| C34.2 | Middle lobe |
| C34.3 | Lower lobe |
| C34.8 | Overlapping lesion of lung |
| C34.9 | Lung, not otherwise specified |

**Footnote:** ICD-O-3 = International Classification of Diseases for Oncology, Third Edition. Source: (1)

1. National Cancer Institute. ICD-O-3 Site Codes: U.S. National Institutes of Health; 2024 [Available from: https://training.seer.cancer.gov/modules_site_spec.html.
